# Distinct gut microbial communities and functional predictions in divergent ophiuroid species: host differentiation, ecological niches, and adaptation to cold-water habitats

**DOI:** 10.1128/spectrum.02073-23

**Published:** 2023-10-27

**Authors:** Yue Dong, Yixuan Li, Meiling Ge, Tetsuya Takatsu, Zongling Wang, Xuelei Zhang, Dewen Ding, Qinzeng Xu

**Affiliations:** 1 College of Environmental Science and Engineering, Ocean University of China, Qingdao, China; 2 Key Laboratory of Marine Eco-Environmental Science and Technology, First Institute of Oceanography, Ministry of Natural Resources, Qingdao, China; 3 Department of Biology, Hong Kong Baptist University, Hong Kong SAR, China; 4 Faculty of Fisheries Sciences, Hokkaido University, Hakodate, Japan; University of Valencia, Paterna, Valencia, Spain

**Keywords:** cold-water environment, ecological niche, gut microbiota, host differentiation, Ophiuroidea

## Abstract

Gastrointestinal microorganisms play a crucial role in host survival and adaptation, but information on host-specific selection or environmental factors that shape the microbiome in natural populations is limited. In this study, we employed 16S rRNA gene amplicon sequencing to investigate the composition and predicted the functions of gut microbiota of two ophiuroid species (*Ophiura sarsii* and its subspecies *O. sarsii vadicola*) from cold-water habitats across three geographically distant sea areas in the Northern Pacific Ocean. We also explored the potential influence of the host and environment on the microbiota. The two ophiuroids possessed diverse microbial communities, with Proteobacteria being the most dominant phylum in all four groups. *Aliivibrio* was the predominant genus in *O. sarsii* from the Bering Sea, while *Candidatus* Hepatoplasma was the dominant genus in *O. sarsii* from the Japan Sea and *O. sarsii vadicola* from the Yellow Sea. Predicted bacterial functions indicated that most of the pathways with significant differences belonged to metabolism and genetic information processing. Notably, no significant difference was observed between the two coexisting ophiuroid species in the Japan Sea. The high similarity in microbial communities between *O. sarsii* from the Japan Sea and *O. sarsii vadicola* from the Yellow Sea may be attributed to their analogous ecological niche. The host and the environment jointly shape the composition of the gut microbial community. The presence of specific microorganisms supports the ecological success of ophiuroids across diverse environments, providing a foundation for host adaptation to cold-water habitats.

**IMPORTANCE:**

Gastrointestinal microorganisms are critical to the survival and adaptation of hosts, and there are few studies on the differences and functions of gastrointestinal microbes in widely distributed species. This study investigated the gut microbes of two ophiuroid species (*Ophiura sarsii* and its subspecies *O. sarsii vadicola*) in cold-water habitats of the Northern Pacific Ocean. The results showed that a combination of host and environmental factors shapes the intestinal microbiota of ophiuroids. There was a high similarity in microbial communities between the two groups living in different regions, which may be related to their similar ecological niches. These microorganisms played a vital role in the ecological success of ophiuroids as the foundation for their adaptation to cold-water environments. This study revealed the complex relationship between hosts and their gut microbes, providing insights into the role they play in the adaptation and survival of marine species.

## INTRODUCTION

The gastrointestinal microbiota is essential for the development and maintenance of intestinal health, as well as protection against harmful pathogens, ultimately contributing to the survival of the host (1, 2). Gastrointestinal microbes, by participating in the host’s metabolic processes, can assist the host in obtaining energy more effectively from food (3, 4). This leads to ecological isolation and differentiation from species that do not harbor symbiotic microorganisms (5, 6). They also play a vital role in the maturation of the host’s local adaptation and development (7, 8). Pronounced interpopulation and interindividual variations in the gut microbial communities are observed in vertebrates and invertebrates, with contributions from endogenous factors (age, sex, and genotype) (9, 10) and exogenous factors (diet, habitat, and environment) (11 – 13).

Echinoderms serve important roles in marine ecosystems as predators and prey (energy cycling) and ecosystem engineers (habitat-forming) and provide economic value to human communities (via commercial harvest) (14). 16S rRNA gene amplicon sequencing has been widely used in the analysis of intestinal microorganisms of echinoderms, such as the sea cucumber (15, 16), sea star (17), and sea urchin (18, 19). A study on Antarctic echinoids with deposit feeding showed that the composition of its gut microbiota was mostly driven by the host type and, to a lesser extent, by the population location (20). Ophiuroidea (brittle stars), the largest class of echinoderms, are distributed in various habitats from intertidal zones to the deep sea (21 – 23). As a significant epifaunal group, ophiuroids have important ecological functions in material circulation and energy conversion in the benthic boundary layer (24) and are vital components of the marine calcium cycle and calcium reservoir (25). Ophiuroids occupy a vital position in benthic food webs, serving as a critical food source for benthic fish, starfish, and crabs (26, 27). The microbial community structure and predictive function in the gut of ophiuroids have been studied extensively in the Yellow Sea. The study reported two feeding types—suspension feeding/herbivores and scavenger/carnivores—and their respective microbial communities, providing insights into the complex interactions between the host and their gut microbiota (28).

*Ophiura sarsii*, as a common circumpolar species, is widely distributed in soft sediments throughout the North Pacific, North Atlantic, and Arctic oceans (29). This genus, as a carnivore feeding type, is a trophic generalist feeding on dead organisms and small animals (usually dead), including crustaceans, mollusks, annelids, and amphipods (30, 31). The assemblages of ophiuroid populations are a common phenomenon in many habitats (32), and they can transfer materials from the surface sediment to higher trophic levels (33, 34), thereby enhancing the process of benthic-pelagic coupling in the sea floor (23). The subspecies *O. sarsii vadicola* is a dominant benthic species from Funka Bay in the Japan Sea and the Yellow Sea Cold Water Mass (YSCWM) (35 – 37). Relevant studies on the evolution and ecology of *Ophiura* spp. have been reported (38, 39). Our previous population genetic studies reported genetic differentiation between *O. sarsii* and the subspecies *O. sarsii vadicola* and showed that there were significant genetic differences (40). These two species were considered as typical models to study species differentiation, but the composition and diversity of their gut microbial communities are still unclear. Since the ecology and evolution of the host are closely relevant to the composition of its gut microbiota (41), it is unclear whether the differentiation of the two ophiuroid hosts affects the microbial composition of their gut microbiota.

In this study, the gut microbiota community of two ophiuroids from three sea areas in typical cold-water habitats in the Pacific Arctic region was analyzed using 16S rRNA sequencing. Our study focused on the gut microbiota, including the compositional structure, predictive functional capabilities, dominant genera, and explored the impact of biological and environmental factors on the composition and structure of these microorganisms.

## RESULTS

### Composition and diversity

**Fig 1:**
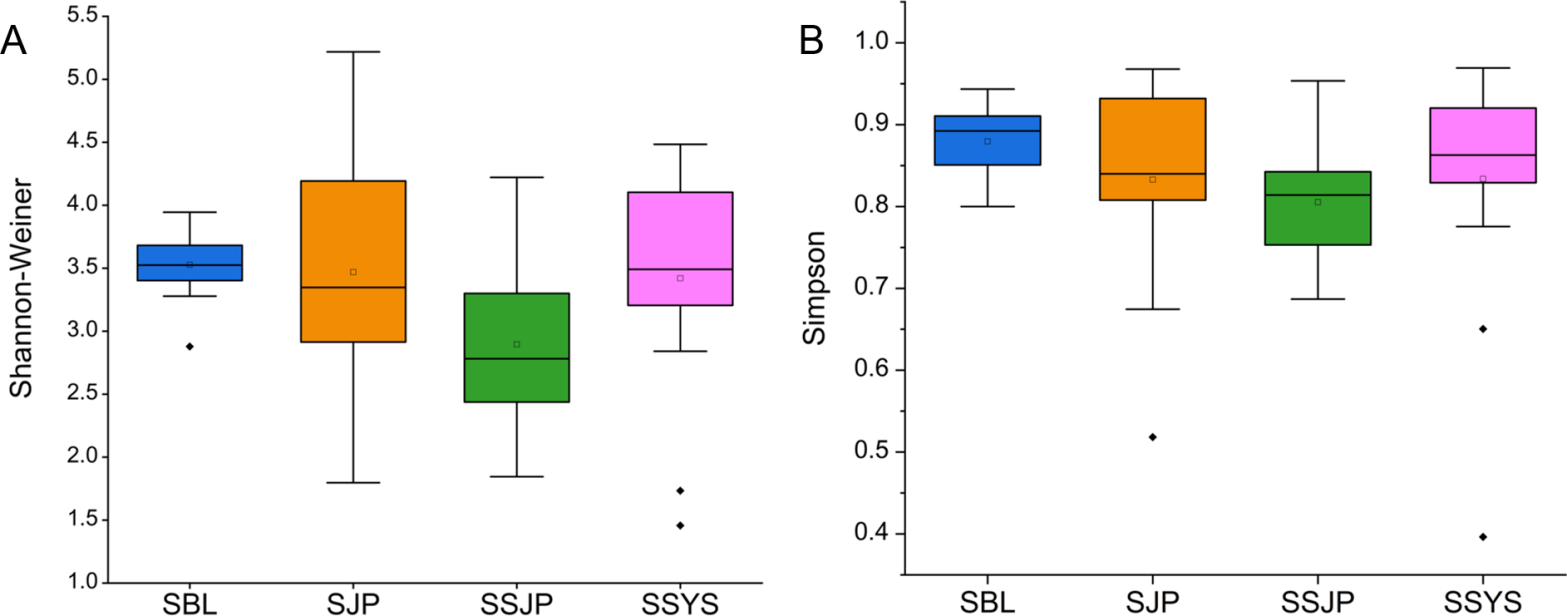
Alpha diversity was calculated using (**A**) the Shannon-Weiner index and (**B**) the Simpson index. Statistical analysis was conducted on alpha diversity using Mann-Whitney *U* tests, *P* > 0.05. *O. sarsii* in the Bering Sea (SBL), *O. sarsii* in Funka Bay (SJP), *O. sarsii vadicola* in Funka Bay (SSJP), and *O. sarsii vadicola* in the Yellow Sea (SSYS).

In total, 6,386,699 high-quality (>Q30) reads were filtered from 6,877,421 raw reads obtained from 56 gut content samples. A total of 19,966 amplicon sequence variants (ASVs) were retained at the 99% similarity level (Data S1). Among these ASVs, 19,860 (99.5%) ASVs were taxonomically assigned to bacterial, and 81 ASVs were archaeal. Only one ASV was eukaryote, and the remaining 24 ASVs were taxonomically unassigned. At the ASV level, alpha diversity analysis was performed on the brittle stars using two indexes (Fig. 1A and B). Shannon-Weiner and Simpson index results showed lower values in *O. sarsii vadicola* from Funka Bay (SSJP, 2.92 and 0.81, respectively), and their values were similar in the two groups of *O. sarsii* in Funka Bay and *O. sarsii vadicola* in the Yellow Sea (3.46, 3.42 and 0.83, 0.83, respectively).

**Fig 2:**
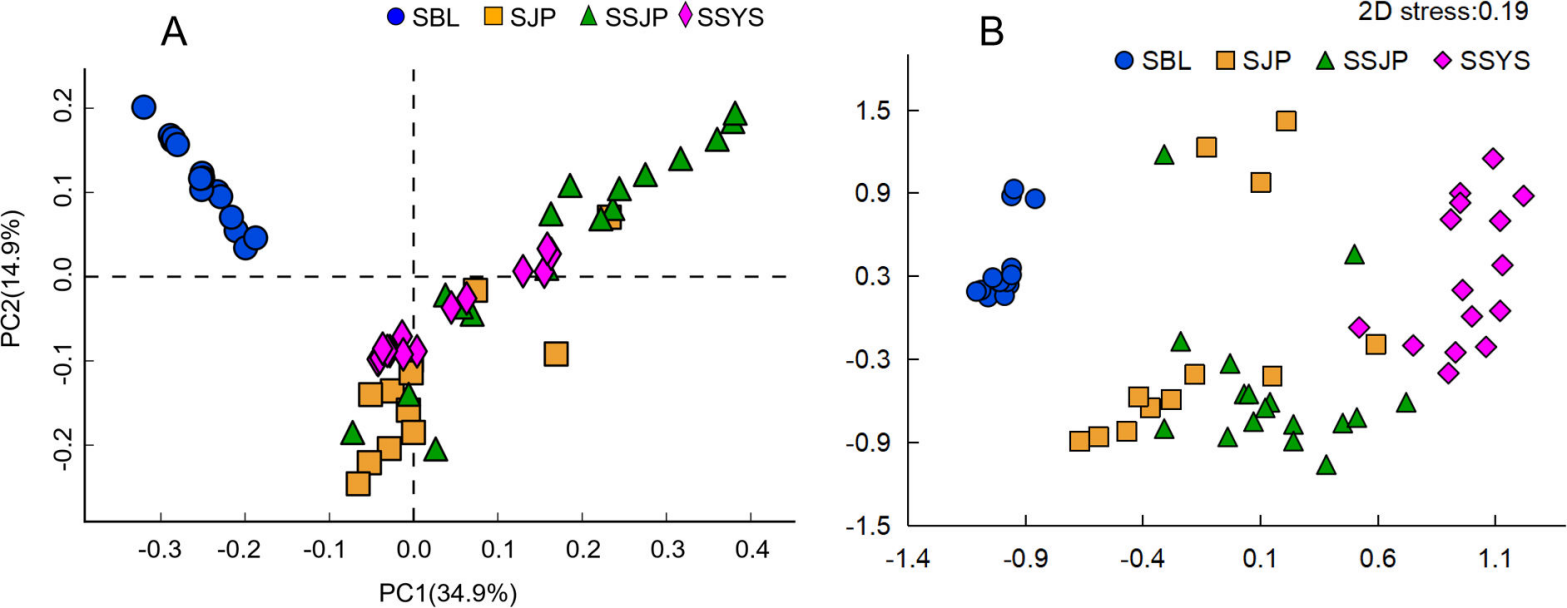
Principal component analysis (PCA) (**A**) and multidimensional scaling (MDS) (**B**) analysis of gut microbiota of ophiuroids at the ASV level. *O. sarsii* in the Bering Sea (SBL), *O. sarsii* in Funka Bay (SJP), *O. sarsii vadicola* in Funka Bay (SSJP), and *O. sarsii vadicola* in the Yellow Sea (SSYS).

At the ASV level, the results of principal component analysis (PCA) showed that the cumulative sum of the values was 49.8%. The two groups (SBL and SSYS) displayed mutual aggregation with good repeatability, while the two groups of ophiuroids from Funka Bay (SJP and SSJP) represented outliers (Fig. 2A). The stress value of multidimensional scaling (MDS) was 0.19 (Fig. 2B). The results also indicated that groups SBL and SSYS clustered separately, while the two groups from Funka Bay did not exhibit clear separation.

**Fig 3:**
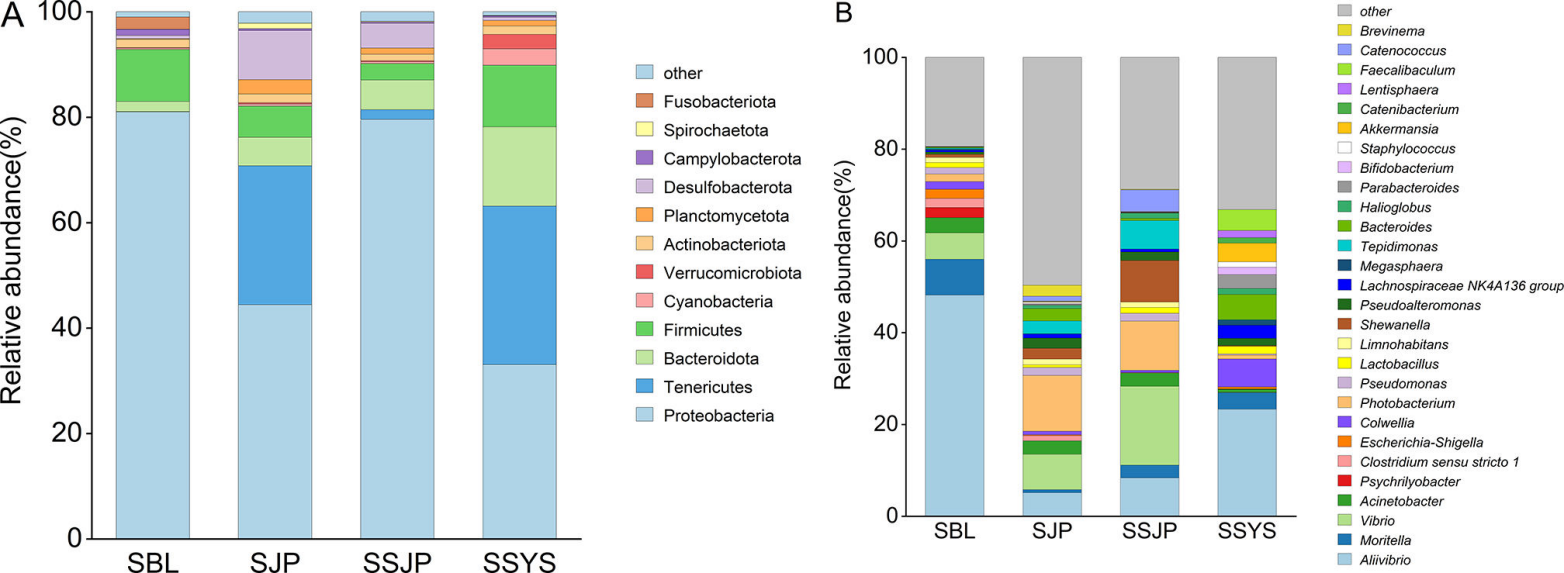
Relative abundance of gut microbiota at the phylum level (**A**) and genus level (**B**) in ophiuroids from the three sea areas. Groups with <1.0% abundance were summarized as other. *O. sarsii* in the Bering Sea (SBL), *O. sarsii* in Funka Bay (SJP), *O. sarsii vadicola* in Funka Bay (SSJP), and *O. sarsii vadicola* in the Yellow Sea (SSYS).

Overall, representatives of 60 phyla, 121 classes, 308 orders, 465 families, 1,172 genera, and 385 species were annotated in gut samples of ophiuroids. Dominant phyla were shared among the groups, and their relative abundance changed with hosts and sea areas (Fig. 3A). Proteobacteria was the most dominant phylum in all populations, followed by Firmicutes, Tenericutes, Bacteroidota, and Desulfobacterota in different groups (Fig. 3A). The relative abundance of Proteobacteria (81.02%, 44.45%), Tenericutes (0.04%, 26.67%), and Bacteroidota (1.93%, 5.43%) showed significant differences in the two groups of *O. sarsii* (SBL and SJP, *P* < 0.05). In addition to these three phyla, Firmicutes (2.50%, 11.62%) were also displayed significantly different in *O. sarsii vadicola* (SSJP and SSYS, *P* < 0.05). Notably, among the two species of brittle stars in Funka Bay, significant differences were also found in the relative abundance of Proteobacteria and Bacteroidota (SJP and SSJP, *P* < 0.05).

At the genus level, as the unique genus of Tenericutes, *Ca*. Hepatoplasma was the most enriched group in the gut microbiota of groups SJP (43.77%) and SSYS (44.92%), whereas it was few in groups SBL (0.04%) and SSJP (4.73%). Regarding the high abundance of this genus, relative abundance analysis was reconducted at the genus level after discarding the ASVs of this genus. The results showed that *Aliivibrio* was the shared genus in all four groups of brittle stars (Fig. 3B). Its relative abundance was 48.21%, 5.13%, 8.37%, and 23.34% in SBL, SJP, SSJP, and SSYS, respectively, with group SBL having the highest abundance. In addition, *Moritella* (7.81%) and *Vibrio* (5.72%) were the dominant genera of *O. sarsii* in the Bering Sea. *Photobacterium* (12.17%, 10.72%) and *Vibrio* (7.71%, 17.18%) were the dominant genera of Funka Bay, with two more genera, *Shewanella* (9.07%) and *Tepidimonas* (6.20%), in *O. sarsii vadicola* (SSJP). Different from the other three groups, *Colwellia* (6.11%) and *Bacteroides* (5.55%) were the dominant genera of *O. sarsii vadicola* in the Yellow Sea (SSYS).

**Fig 4:**
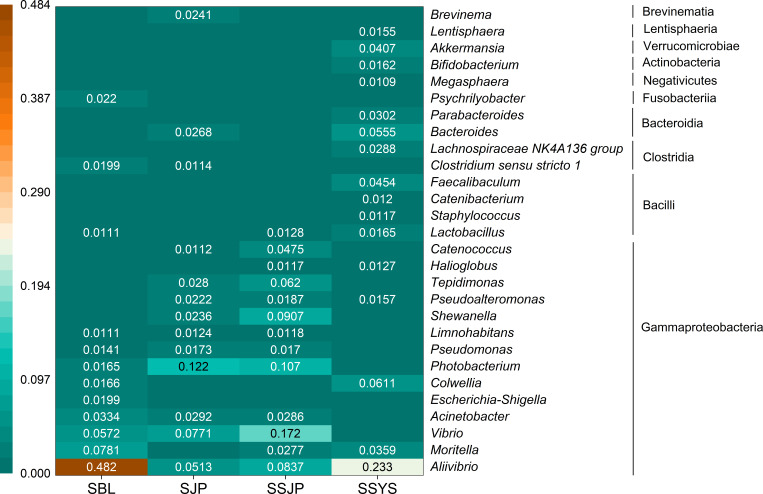
The heatmap of the relative abundance of gut microbiota at the genus level (>1.0%) in ophiuroids from three sea areas. *O. sarsii* in the Bering Sea (SBL), *O. sarsii* in Funka Bay (SJP), *O. sarsii vadicola* (SSJP) in Funka Bay, and *O. sarsii vadicola* in the Yellow Sea (SSYS).

The results of the heatmap for the four ophiuroid groups showed that Gammaproteobacteria was the dominant class (Fig. 4). The significance analysis of the relative abundance of dominant genera revealed that there were significant differences (*P* < 0.05) between *O. sarsii* (SBL and SJP) and its subspecies (SSJP and SSYS). Besides, no significant differences were observed between the two ophiuroid groups in Funka Bay (SJP and SSJP, *P* > 0.05). These results showed the diversity in the gut microbial communities of ophiuroids. Between the two groups of *O. sarsii* (SBL and SJP), all the genera with significant differences belonged to class Gammaproteobacteria, except for *Psychrilyobacter* (class Fusobacteriia) and the CL500-29 marine group (class Acidimicrobiia, Table S1). Similarly, for *O. sarsii vadicola* (SSJP and SSYS), more than half of the genera showed significant differences that belonged to Gammaproteobacteria, with some classified as Vibrionaceae (Table S2).

Additionally, we also analyzed the microorganisms occurring in both ophiuroids and sediments, and the results presented that the dominant phyla and genera differed between the brittle stars and their respective environments (Fig. S1A and B). Moreover, the dominant phyla among the three sediment groups were distinct. Proteobacteria was the dominant phylum in the Bering Sea, while Chloroflexi and Caldatribacteriota were the dominant phyla in the Japan Sea. In the Yellow Sea, Proteobacteria and Planctomycetota were the primary phyla. At the genus level, there was no shared dominant bacterial genus among the three sediment groups, with *Woeseia* being abundant only in the Bering Sea and Yellow Sea.

### Predictive function of the microbiomes

**Fig 5:**
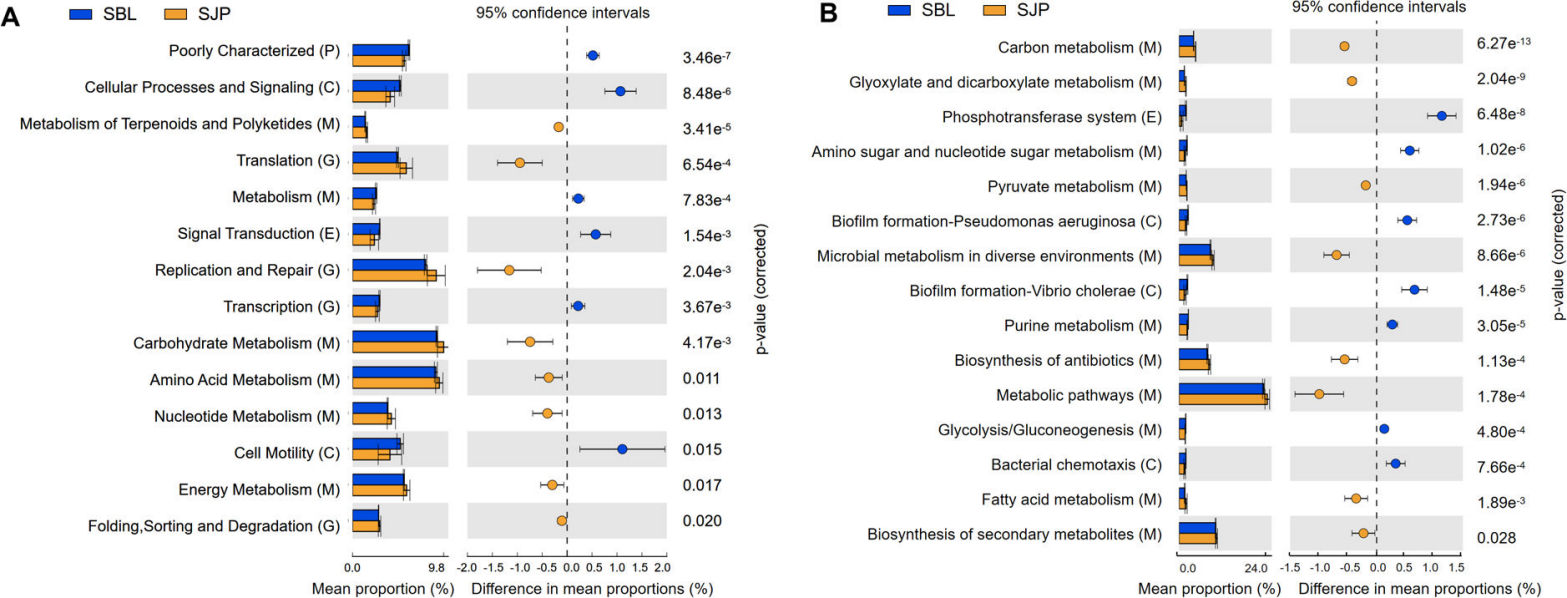
The significant expression pathways based on (**A**) PICRUSt2 and (**B**) Tax4Fun2 analyses of the gut microbial community between *O. sarsii* at Kyoto Encyclopedia of Genes and Genomes level 2. *O. sarsii* in the Bering Sea (SBL) and *O. sarsii* in Funka Bay (SJP).

Phylogenetic Investigation of Communities by Reconstruction of Unobserved States (PICRUSt2) and Tax4Fun2 analyses annotated 29 and 19 pathways in the second classification (>1.0%) in brittle stars, respectively (Data S2 and S3). In *O. sarsii*, 14 and 15 pathways showed significant differences between the Bering Sea group and the Funka Bay group (*P* < 0.05), and most of the pathways belonged to metabolism and genetic information processing (Fig. 5A and B).

Similarly, *O. sarsii vadicola* also exhibited significant differences in Funka Bay and the Yellow Sea (SSJP and SSYS) in 17 and 9 pathways using PICRUSt2 and Tax4Fun2 analyses, respectively. More metabolism-related pathways are annotated in *O. sarsii vadicola* compared to those of species groups (Fig. S2A and B). We also compared the two ophiuroid groups coexisting in Funka Bay (SJP and SSJP), and the results of PICRUSt2 revealed significant differences in 12 pathways, also relevant to metabolism and genetic information processing (Fig. S3). However, the results of Tax4Fun2 analysis showed no significant differences between these two groups.

Furthermore, we also compared the microbiome of *O. sarsii* in Funka Bay (SJP) and *O. sarsii vadicola* in the Yellow Sea (SSYS), and the PICRUSt2 results showed that only three pathways were significantly different, including xenobiotic biodegradation and metabolism, lipid metabolism, and transcription (Fig. S4).

### Phylogenetic evolution of dominant bacteria

**Fig 6:**
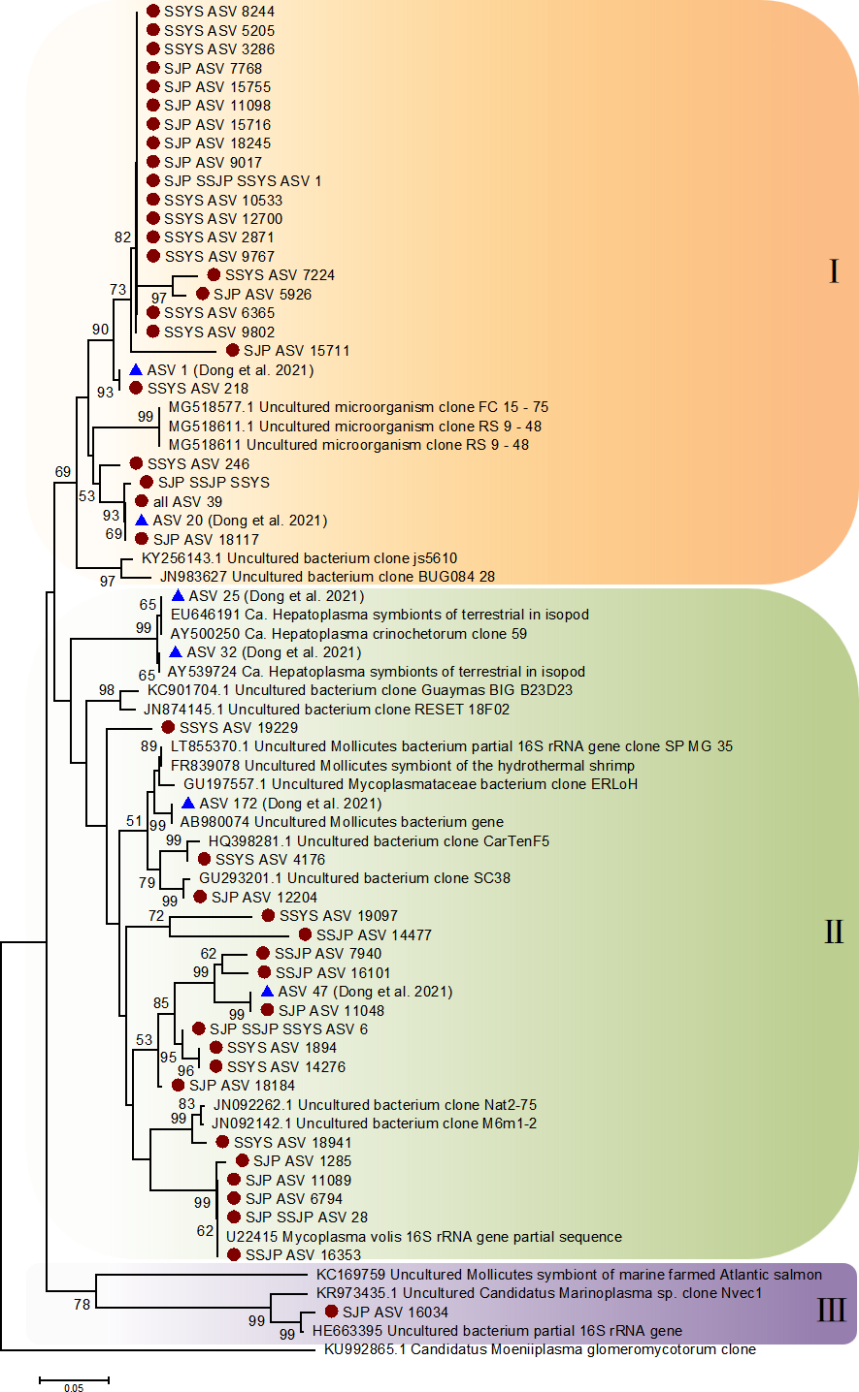
Phylogenetic analysis of *Ca.* Hepatoplasma based on 16S rRNA sequences. The node bootstrap value below 50 is not shown. Sequences from the four groups in this study are marked with red circles, while the ASVs of the previous study are marked with blue triangles.

The numbers of ASVs annotated as *Ca*. Hepatoplasma in the four ophiuroid groups were 1, 22, 10, and 22, respectively (Fig. 6). ASV39 was shared by all four groups, while three sequences (ASV1, ASV6, and ASV68) were present in both Funka Bay and the Yellow Sea. ASV28 and ASV935 were observed only in Funka Bay. The sequences were clustered into three clades. Both Clade I and Clade II contained reference sequences from Dong et al. (28). Clade I was mainly a group of *O. sarsii* in Funka Bay and *O. sarsii vadicola* in the Yellow Sea (SJP and SSYS). Clade II included the groups from Funka Bay and a small amount of sequences from group SSYS, and Clade III had only one sequence from group SJP clustered with the reference sequences.

**Fig 7:**
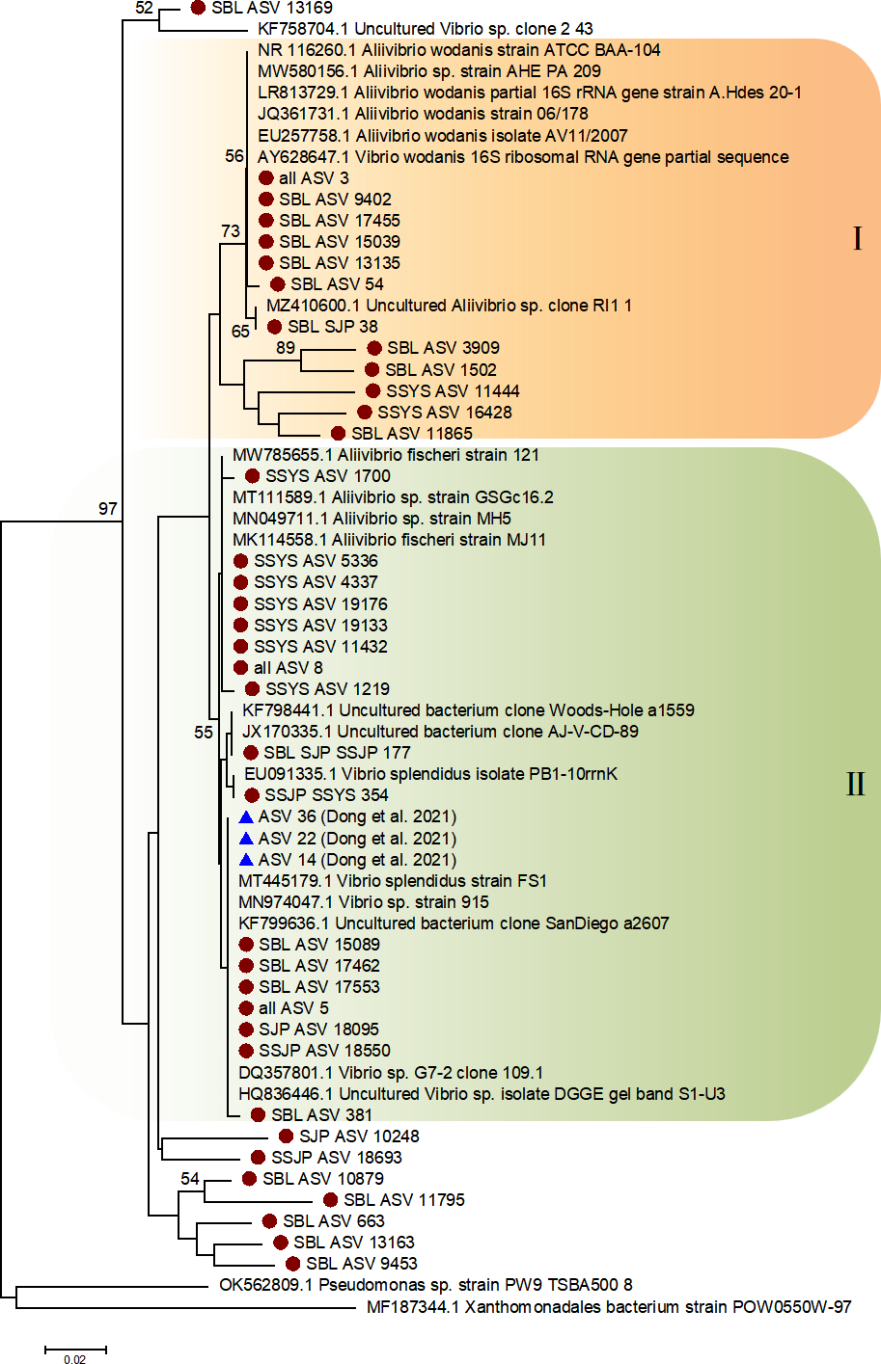
Phylogenetic analysis of *Aliivibrio* based on 16S rRNA sequences. The node bootstrap value below 50 is not shown. Sequences from the four groups in this study are marked with red circles, while the ASVs of the previous study are marked with blue triangles.

We also conducted a phylogenetic analysis of *Aliivibrio,* and the numbers of ASVs annotated as the genus in the four ophiuroid groups were 23, 6, 7, and 14, respectively (Fig. 7). Three sequences (ASV3, ASV5, and ASV8) were shared by all four groups. These sequences were mainly clustered into two groups. Clade I was the aggregation of groups SBL and SSYS. Clade II contained a variety of sequences, including those ASVs from groups SBL and SSYS, shared ASV sequences, and reference sequences from Dong et al. (28).

## DISCUSSION

### The gut microbiota composition of ophiuroids

In this study, we compared the gut microbial community structure of *O. sarsii* and its subspecies *O. sarsii vadicola* in three Pacific Arctic regions (Bering Sea, Funka Bay, and Yellow Sea) to investigate how their gut content is potentially shaped by the host and environment. We selected the V4 region for amplification and obtained the dominant intestinal phyla, including Proteobacteria, Tenericutes, Firmicutes, and Bacteroidetes, which were consistent with our previous study by the V3-4 region using the same database (28). These findings suggested that despite variations in the gut microbiome profile, a core microbiome seemed to exist in ophiuroids. This could be hypothesized as a result of the co-evolution of beneficial gut microbes with their hosts (42). Due to the long storage time of alcohol samples from Funka Bay, high-quality V3-4 region sequences could not be obtained. Therefore, we selected the V4 region for amplification in this study. Consequently, we also identified more genera with low abundance and obtained four times the number of unique genera compared to our previous study using the V3-4 region (Table S3), demonstrating that the V4 region was also better able to capture the overall microbial diversity. In large-scale microflora investigation studies, such as the Earth Microbiome Project, American Gut Project, and global topsoil microbiome, the V4 region was the most widely used and recognized test area in international research (43, 44).

In our study, we found a wide variety of microorganisms present in the host gastrointestinal system, which play a crucial role in host physiology and ecology. As the dominant phyla, Firmicutes and Bacteroidota were known to participate in carbohydrate and polysaccharide digestion (45, 46). Tenericutes was the second dominant phylum in ophiuroids from Funka Bay, relevant to polysaccharide digestion in the vertebrate microbiota (47, 48). By using PICRUSt2 and Tax4Fun2 (Fig. 5), we were able to predict carbohydrate metabolism pathways and amino acid metabolism, potentially essential for the digestion of starch and protein. Notably, *Vibrio*, *Photobacterium*, and *Shewanella* were found to be the most abundant genera in SSJP (Fig. 3B), suggesting that they likely provided the majority of the essential fatty acids for brittle stars’ survival. Studies have shown that these genera can use fatty acid synthase/polyketide synthase (FAS/PKS)-type enzyme systems to synthesize polyunsaturated fatty acids from scratch through the anaerobic pathway (49 – 51). The gut microbiota of *O. sarsii* specimens, collected from the Bering Sea in the subarctic areas, contained a notably higher abundance of *Psychrilyobacter atlanticus* compared to other groups. Previous research has demonstrated that *P. atlanticus* was a crucial substance for protein degradation in subarctic marine sediments (52). Additionally, members of this genus have been shown to break down necrotic material from whole spirulina (53, 54). As a result, it is hypothesized that this genus may be involved in the degradation of protein and lipid macromolecules within the food consumed by ophiuroids.

The genus *Aliivibrio*, a member of Gammaproteobacteria, was the predominant microbe that occurred in the gut of brittle stars from both the Bering Sea and the Yellow Sea. Despite that, *Aliivibrio* was also the dominant genus among stomach microorganisms of fish (55). This genus was common in cold-water environments, with some species exhibiting cold adaptability, and several strains in this genus have been exploited as potential hosts for heterologous expression of cold-active enzymes (56, 57). The Bering Sea, situated in a high-latitude region, experiences cold water masses during the summer season, with an average temperature of approximately 0°C (58). *O. sarsii vadicola* inhabits areas characterized by the Yellow Sea Cold Water Mass, with temperatures consistently remaining below 6°C for many years (59). The above two cold-water environments provide cold-water habitats for *Aliivibrio. Ophiura* was known to be a scavenger that preys on carrion (60), and *Aliivibrio* can be identified in decaying materials in the ocean (61, 62), so this genus may also be passively introduced into the gut through the predatory behavior of brittle stars. These factors may explain the high abundance of the genus in brittle stars. As a carrier of cold enzymes, *Aliivibrio* may contribute to the development of ophiuroids and their adaptation to cold-water environments. Further investigation using metagenomic tools is needed to comprehensively understand the role of this genus in hosts from these ecosystems.

In our study, *Ca*. Hepatoplasma was detected in the gut microbes of ophiuroids by both V3-4 region and V4 region methods (28). *Ca*. Hepatoplasma, with high abundance in SJP and SSYS, was commonly found in some amphipods (63), terrestrial isopods (64), and echinoderms (65). It has been identified as a mycoplasma-like symbiont with a number of genes responsible for nutrient absorption (66), suggesting that it may be essential for the survival of hosts under low nutrient conditions (67). The large assemblages of *Ophiura* spp. may have a negative impact on population development by causing food scarcity. On the other hand, a high abundance of bacteria can help the host survive in nutritionally deprived environments, highlighting the role of microorganisms in host environment adaptation.

### Biotic and abiotic factors influenced the gut microbiota

The relationship between hosts and microbes is a prominent area of current research (68 – 70). The host phylogenetic relationship was considered an important factor in determining gut microbial diversity (71, 72). *Ophiura* spp., a common species in the Pacific Arctic region, exhibited differentiation between species and subspecies (38, 40). In our previous study, they were divided into four geographical populations across three sea areas (39), including *O. sarsii* in the Bering Sea (SBL), *O. sarsii* in Funka Bay (SJP), *O. sarsii vadicola* (SSJP) in Funka Bay, and *O. sarsii vadicola* in the Yellow Sea (SSYS). This property makes them a valuable model for studying the effects of invertebrate hosts on microorganisms and elucidating the complex dynamic relationships between hosts and microbiomes. Interestingly, in line with the trend of host geographic differentiation, significant differences in the microbial communities were observed between species groups (SBL and SJP) and subspecies groups (SSJP and SSYS). Therefore, it was speculated that the differentiation of brittle stars may have an impact on the microorganisms residing in their gastrointestinal tract.

However, no significant difference was found in the gut microbiota between the species and the subspecies (SJP and SSJP) that have already differentiated in the Japan Sea. We proposed that the gastrointestinal microbial community was influenced by both host differentiation and environmental factors (biotic factors and abiotic factors), with the environment potentially playing a more important factor compared to the host (73 – 75). Since the two species coexisted in the same environment, it was likely that environmental factors were the primary drivers of the similarities in their microbial communities. Previous studies have demonstrated that the composition of the intestinal microbiota in healthy individuals was mainly determined by environmental factors, with host genetics playing a minor role in shaping the microbiota composition (76). Similar findings have also been reported in studies on the intestinal microbiota of fish and mice (77, 78).

Numerous studies have demonstrated that temperature could influence the gut microbiota (79, 80). While the three sampling sites in this study were situated in the Northern Pacific cold-water area, differences in the internal environments and water temperatures were evident. The average temperature of the YSCWM in the North Yellow Sea has consistently remained below 6°C for many years (59). Funka Bay had a temperature of around 9°C at a depth of 100 m (https://ds.data.jma.go.jp/kaiyou/data/shindan/c_1/jun_NK/kaikyo_NK.html). The Bering Sea, located at a high latitude, exhibited the lowest water temperature. Under varying water temperature conditions, significant differences were observed among the gut microorganisms of ophiuroids. Notably, various marine psychrophilic bacteria, such as *Colwellia*, *Shewanella*, *Aliivibrio*, and *Moritella*, exhibited distinct distribution patterns across each group.

Additionally, we also conducted an analysis to compare the intestinal microorganisms of the ophiuroids with the surface sediment microbes present in the research sea areas (Fig. S1A and B). The results revealed a relatively low similarity between the three groups of sediment microbes, implying that the benthic environment varied across the sea areas. However, due to the limited publicly available microbial data on surface sediments in Funka Bay, we had to rely on sediment data obtained at a depth of 1.4 m near the sampling station (81). Owing to variations in the internal environment of the cold-water habitats (Pacific Arctic regions), the gut microbes of hosts from each region also differed. Consequently, further investigations using metagenomics and other methods are required to explore the biological connectivity and distinctions between hosts and their gastrointestinal microbes.

### Effects of niche action on microorganisms

As previously mentioned, there were significant differences in the gut microbiota between the species (*O. sarsii*, SBL and SJP) and the subspecies (*O. sarsii vadicola*, SSJP and SSYS), respectively. However, despite their host phylogenetic differentiation, groups SJP and SSYS exhibited more similar gut microbial compositions. Both groups displayed a high abundance of *Ca*. Hepatoplasma, potentially reflecting host adaptation to the environment as large populations. Moreover, the prediction function of microbiomes between *O. sarsii* (SJP) and *O. sarsii vadicola* (SSYS) also verified this possibility, with most pathways showing no significant differences. The high similarity of microbiota and functional prediction indicated that the two species may occupy the same ecological niche.

The genus *Ophiura* was typically deposit feeders, consuming organic detritus, benthic microalgae, and benthic invertebrates (82, 83). In the dense beds of Funka Bay, *O. sarsii* primarily fed on shrimp species and small crustaceans, such as cumaceans and benthic amphipods (83). Similarly, amphipods and bivalve mollusks were also found in *O. sarsii vadicola* from the Yellow Sea. Moreover, both ophiuroids were dominant species in their respective environments. In Funka Bay, *O. sarsii* is the dominant species, accounting for over 80% of the *Ophiura* spp. on the seabed (37), while the other remaining ophiuroid was the subspecies *O. sarsii vadicola* (H. Izumiura, unpublished data). Meanwhile, *O. sarsii vadicola* was the dominant species in the benthic environment of the YSCWM due to its large population (84). Microorganisms are influenced not only by the geographical environment but also by niche processes in the unified environment (85). A study showed that the ecological niche shaped the cichlid fish gut microbiota in Central American and African lakes (86).

The two species with similar feeding types are both carnivorous and were dominant species in their respective benthic habitats, so it was speculated that they have the same dietary niche. Dietary niches, as a primary influence, impact the gut microbiota by providing specific nutrients and substrates that select certain microbial species, thus affecting overall community structure (86), as observed in the cichlid species complex (86, 87). Our previous studies revealed significant differences in gut microbes among ophiuroids with varying feeding types, while the gut microbes of two carnivorous ophiuroids (*Stegophiura sladeni* and *O. sarsii vadicola*) were similar (28).

In this study, we found distinct microbial communities in the two ophiuroid species, influenced by both host differentiation and environmental factors. The similarity between *O. sarsii* from Funka Bay and *O. sarsii vadicola* from the Yellow Sea may be due to their similar ecological niches. Our findings highlight the interplay between host variation, environmental factors, and microbial communities in cold-water habitats.

## MATERIALS AND METHODS

### Sample collection

**Fig 8:**
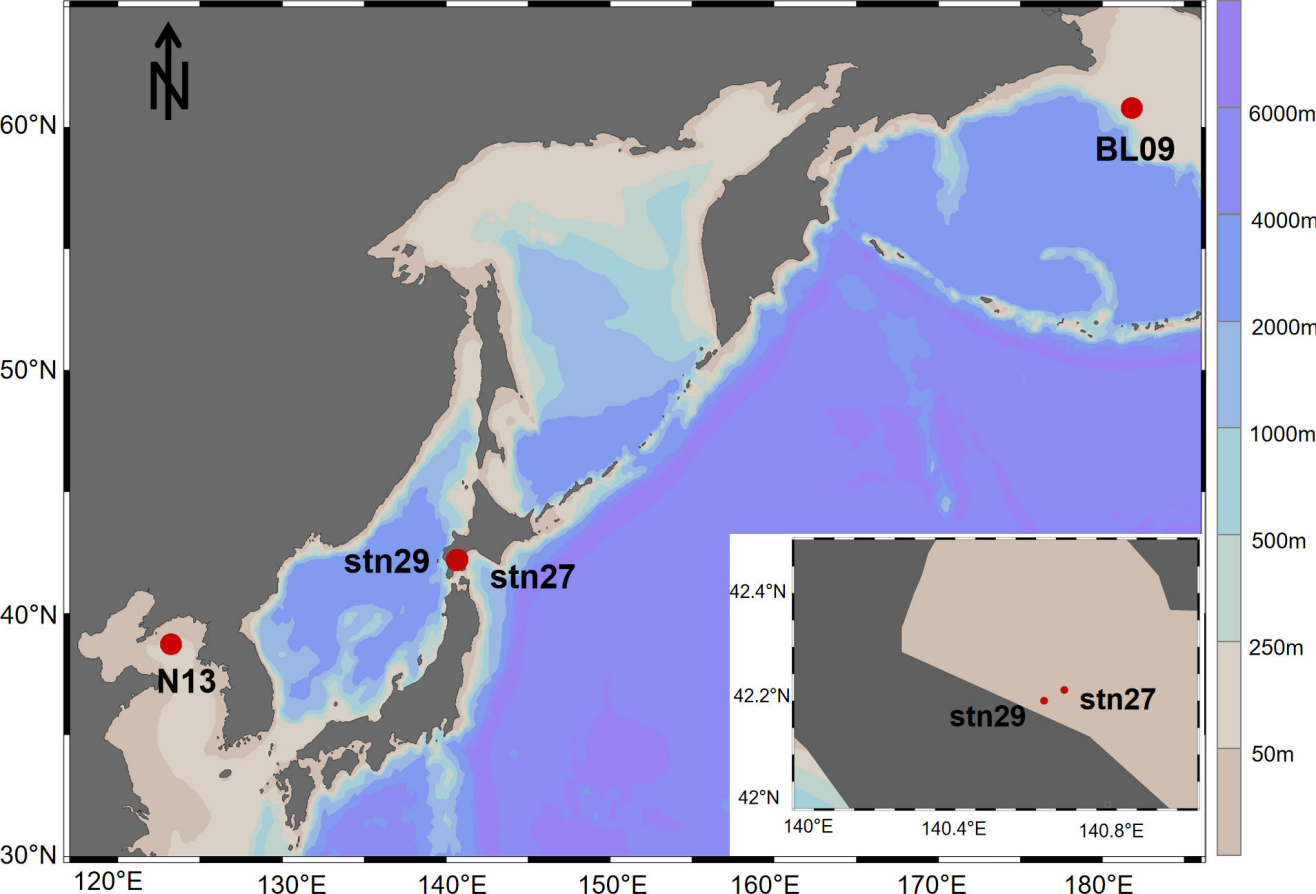
Sampling sites of ophiuroids from the Bering Sea, Funka Bay in Japan, and the Yellow Sea. The map was created using Ocean Data View (ODV, https://odv.awi.de/).

Ophiuroid specimens were collected by bottom trawling in the above three sea areas: *O. sarsii* in the Bering Sea (SBL, 15 individuals) and *O. sarsii vadicola* in the Yellow Sea (SSYS, 15 individuals) from 2019 aboard the Xiangyanghong 01 research vessel and 2021 Lan Hai 101 sharing cruise, respectively (Fig. 8). *O. sarsii* (SJP, 12 individuals) and *O. sarsii vadicola* (SSJP, 17 individuals) in Funka Bay, Japan, were collected with a bottom sledge net by T/S Ushio-maru of Hokkaido University (Table 1). To minimize potential distress, all specimens were immediately preserved in 95% ethanol after collection and stored at −20°C. Our sampling methodology aligns with ethical standards for invertebrate research. After the cruises, specimens were transferred to the First Institute of Oceanography, Ministry of Natural Resources, for further analysis. To reduce contamination by environmental bacteria, the ophiuroids were rinsed with Milli-Q water before dissection. The oral shield was removed, and the gut contents were sampled under a stereomicroscope.

**TABLE 1:** Sampling sites of *O. sarsii* and *O. sarsii vadicola* from the Bering Sea, Funka Bay in Japan, and the Yellow Sea

Sample	Area	Sample name	Collection date	Longitude	Latitude
*O. sarsii*	Bering Sea	SBL	2019.08	178.21°W	60.80°N
	Funka Bay	SJP	2019.08	140.67°E	42.22°N
*O. sarsii vadicola*	Funka Bay	SSJP	2019.08	140.62°E	42.20°N
	Yellow Sea	SSYS	2021.04	123.20°E	38.75°N

Sediment samples from the Bering Sea were also collected by a 0.20-m^2^ sterile stainless steel grab sampler (Wildco, FL, USA) in 2019. The sampling wares and centrifuge tubes were first treated with moist heat sterilization. All samples were stored in a freezer at −20°C until DNA extraction was performed.

### Total DNA extraction and 16S rRNA sequencing

We extracted total DNA from the gut contents and three surface sediment samples (Bering Sea), including 59 ophiuroids with two species from three areas, and amplified sequencing using the V4 region of the 16S rRNA gene. In brief, total genomic DNA was isolated using the DNeasy Blood & Tissue Kit (Qiagen, Germany) following the manufacturer’s protocol. DNA quality was assessed using 1.0% agarose gel electrophoresis and a Qubit fluorometer (Thermo Fisher Scientific, Waltham, MA, USA). The V4 hypervariable region of the 16S rRNA gene was amplified using the forward primer 515F (5′-GTGCCAGCMGCCGCGGTAA-3′) and the reverse primer 806R (5′-GTGGACTACHVGGGTWTCTAAT-3′). The PCR mixture contained 1 µL of DNA template (30 ng/µL), 1 µL each of 5 mol/L primers, 3 µL of bovine serum albumin (BSA, 2 ng/µL), 12.5 µL of 2 × Taq Plus Master Mix, and 7.5 µL of ddH_2_O in a total volume of 25 µL. PCR amplification was performed with the following program: pre-denaturation at 94°C for 5 min; 30 cycles of denaturation at 94°C for 30 s, annealing at 50°C for 30 s, and an extension at 72°C for 60 s; and a final extension at 72°C for 7 min. The PCR products were sequenced using an Illumina MiSeq sequencer to produce 300-bp paired-end reads by Allwegene Technology (Beijing, China).

### Data filtration and amplicon sequence variant clustering

The following taxonomic analyses were performed using the R script implemented in the dada2 v1.16.0 package (88) with default parameters. Raw reads were further quality filtered, dereplicated, and merged using the R script plotQualityProfile, filterAndTrim, derepFastq, and mergePairs, respectively. The makeSequenceTable command was employed to construct an ASV table. Potential chimeras were removed using the removeBimeraDenovo command. ASVs were annotated and compared with the Silva SSU rRNA database (version v138.1) and Silva species assignment (v138.1) for taxonomic classification (89) using the assignTaxonomy command.

In this study, we primarily focused on bacterial analysis, and bacterial ASVs were retained, and other ASVs annotated with archaea and fungi and unidentified were discarded. We also conducted a comparative analysis of microbiota composition at the phylum and genus levels between organisms and sediments. 16S rRNA gene sequencing data of sediment were downloaded from published studies in the North Yellow Sea and the Japan Sea (81, 90). The sediment sequencing data were processed in the same way as biological samples.

The functional profiles of the gut microbial communities based on the 16S rRNA gene sequence were annotated using PICRUSt2 and Tax4Fun2 with Kyoto Encyclopedia of Genes and Genomes (KEGG) Orthology (KO) (91). To compare functional differences among samples, the abundances of predicted functional pathways were normalized to relative abundances. The predictive functional pathway of ophiuroid gut microbial communities was analyzed in pairs, including *O. sarsii* from the Bering Sea and Funka Bay (SBL and SJP), *O. sarsii* and *O. sarsii vadicola* from Funka Bay (SJP and SSJP), *O. sarsii vadicola* from Funka Bay and the Yellow Sea (SSJP and SSYS), and *O. sarsii* from Funka Bay and *O. sarsii vadicola* from the Yellow Sea (SJP and SSYS). We used the same method as in the previous article to analyze the significant differences in KOs with Statistical Analysis of Metagenomic Profiles (STAMP).

### Statistical analysis

Sampling stations were mapped using Ocean Data View (ODV) software. After obtaining the taxonomy table, we conducted subsampling of the data as a preliminary step before performing subsequent data analyses, including diversity calculations. The uniformity of sample sequencing was ensured by the method of rarefaction. In this study, we used the minimum abundance value as the rarefaction depth. Alpha diversity statistics were calculated using diversity metrics based on the Simpson index and Shannon-Weiner index. Mann-Whitney *U* tests were used to evaluate the differences among categories in R. The differences in the species abundance of gut microorganisms were analyzed using Welch’s *t*-test. PCA was performed to reflect the differences and distances between samples at the ASV level using Origin 2021 software (92). MDS analysis of the gut microbiota in ophiuroids was performed using PRIMER (v6) and PERMANOVA+ software (93). Additionally, the abundance of KEGG pathways over 1.0% was selected to calculate the ANOVA between two selected ophiuroid groups by using STAMP software (94).

### Phylogenic analysis

As the dominant genera, we filtered the sequences annotated as *Ca.* Hepatoplasma and *Aliivibrio* from the obtained ASV sequences, respectively. These sequences were uploaded to the National Center for Biotechnology (NCBI) for comparison, a BLAST-n search was conducted against the standard database, and the top two sequences with the highest similarity (88%–99%) were downloaded. Then, along with the sequences from Dong et al. (28), we performed a phylogenetic analysis of the two genera based on sequences from the three sources. In addition, sequences of the same family (*Ca*. Hepatoplasma, Mycoplasmataceae) and class (*Aliivibrio*, Gammaproteobacteria) were added as outgroups. The phylogenetic analysis was conducted using the MEGA v7.0 (95) neighbor-joining (NJ) method with the Kimura two-parameter model and 1,000 bootstrap replications.

## Data Availability

All sequence reads generated in this study have been uploaded onto the NCBI GenBank Sequence Read Archive (SRA) database under the BioProject accession number PRJNA913621.

## SUPPLEMENTAL MATERIAL

Supplemental material spectrum.02073-23-s0001.docx Tables S1 to S3; Fig. S1 to S4.

ASM does not own the copyrights to Supplemental Material that may be linked to, or accessed through, an article. The authors have granted ASM a non-exclusive, world-wide license to publish the Supplemental Material files. Please contact the corresponding author directly for reuse.
