# Supplementary material for: Distinct gut microbial communities and functional predictions in divergent ophiuroid species: host differentiation, ecological niches, and adaptation to cold-water habitats
Source: Microbiol Spectr. 2023 Oct 27;11(6):e02073-23. doi: 10.1128/spectrum.02073-23 (PMC10715168; doi:10.1128/spectrum.02073-23)
Supplement: Supplemental material — Tables S1 to S3; Fig. S1 to S4. [file spectrum.02073-23-s0001.docx]

## Supplementary Material

Table S1 Genera with significant differences in gut microorganisms of *O. sarsii* from Bering Sea and Funka Bay (SBL and SJP)

| Genus | SBL | SJP | Class | Family |
| --- | --- | --- | --- | --- |
| *Aliivibrio* | +++ |  | Gammaproteobacteria | Vibrionaceae |
| *Vibrio* |  | ++ |  | Vibrionaceae |
| *Photobacterium* |  | +++ |  | Vibrionaceae |
| *Moritella* | ++ |  |  | Moritellaceae |
| *Shewanella* |  | ++ |  | Shewanellaceae |
| *Escherichia-Shigella* | + |  |  | Enterobacteriaceae |
| *Tepidimonas* |  | + |  | Comamonadaceae |
| *Pseudoalteromonas* |  | + |  | Pseudoalteromonadaceae |
| *Pseudomonas* |  | + |  | Pseudomonadaceae |
| *Psychrilyobacter* | + |  | Fusobacteriia | Fusobacteriaceae |
| *CL*500-29 *marine group* |  | + | Acidimicrobiia | Ilumatobacteraceae |

The character + represents the relative abundance range of a genus. A single + means less than 5.0%, ++ means between 5.0% and 10.0%, and +++ means more than 10.0%.

Table S2 Genera with significant differences in gut microorganisms of *O. sarsii vadicola* from Funka Bay and Yellow Sea (SSJP and SSYS)

| Group | SSJP | SSYS | Class | Family |
| --- | --- | --- | --- | --- |
| *Vibrio* | +++ |  | Gammaproteobacteria | Vibrionaceae |
| *Aliivibrio* |  | +++ |  | Vibrionaceae |
| *Photobacterium* | +++ |  |  | Vibrionaceae |
| *Catenococcus* | + |  |  | Vibrionaceae |
| *Limnohabitans* | + |  |  | Comamonadaceae |
| *Tepidimonas* | ++ |  |  | Comamonadaceae |
| *Colwellia* |  | ++ |  | Colwelliaceae |
| *Pseudomonas* | + |  |  | Pseudomonadaceae |
| *Acinetobacter* | + |  |  | Moraxellaceae |
| *Akkermansia* |  | + | Verrucomicrobiae | Akkermansiaceae |
| *Bacteroides* |  | ++ | Bacteroidia | Bacteroidaceae |
| *Parabacteroides* |  | + |  | Tannerellaceae |
| *Sva*0081 *sediment group* | + |  | Desulfobacteria | Desulfosarcinaceae |
| *Lentisphaera* |  | + | Lentisphaeria | Lentisphaeraceae |
| *hgcI clade* | + |  | Actinobacteria | Sporichthyaceae |
| *Faecalibaculum* |  | + | Bacilli | Erysipelotrichaceae |

The character + represents the relative abundance range of a genus. A single + means less than 5.0%, ++ means between 5.0% and 10.0%, and +++ means more than 10.0%.

Figure S1 Taxonomic abundance of gut microbiota at the phylum level (A) and genera level (B) in ophiuroid species and sediments from the three sea areas. In the phylum, other represents the sum of taxa with an abundance of less than 1.0%. Similarly, in the genus, other refers to the sum of all groups except for the top 5 most abundant genera. *O. sarsii* in Bering Sea (SBL), *O. sarsii* in Funka Bay (SJP), *O. sarsii vadicola* (SSJP) in Funka Bay, *O. sarsii vadicola* in Yellow Sea (SSYS), sediment in Bering Sea (SedBL), sediment in Japan Sea (SedJP), sediment in Yellow Sea (SedYS)


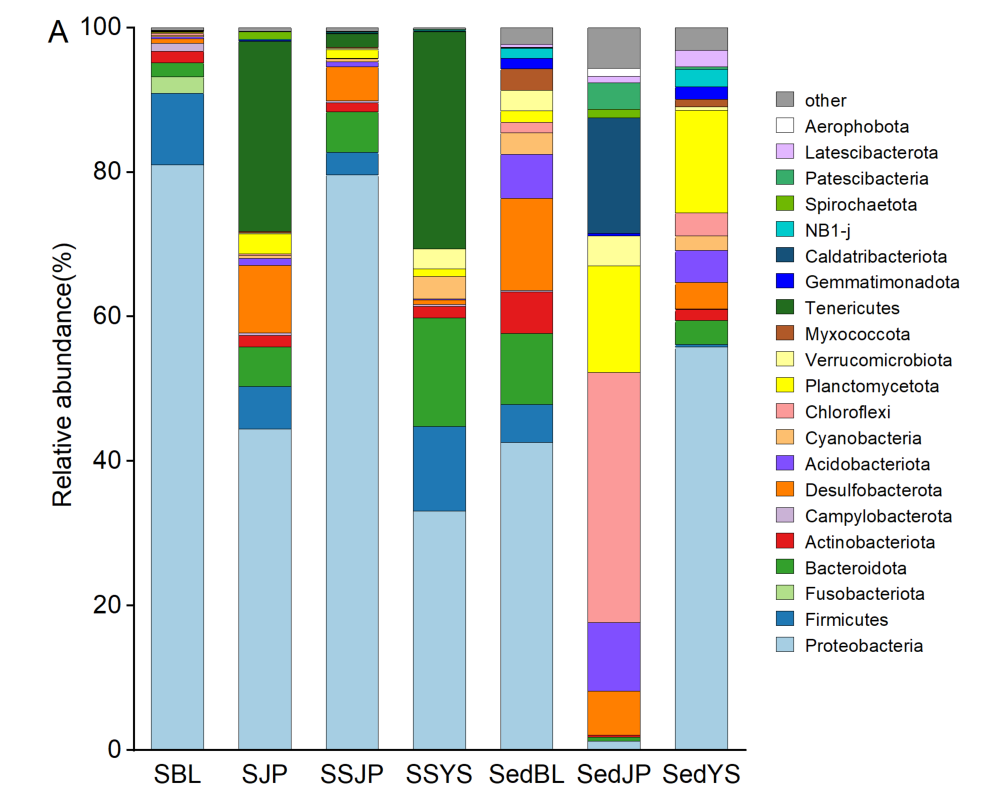


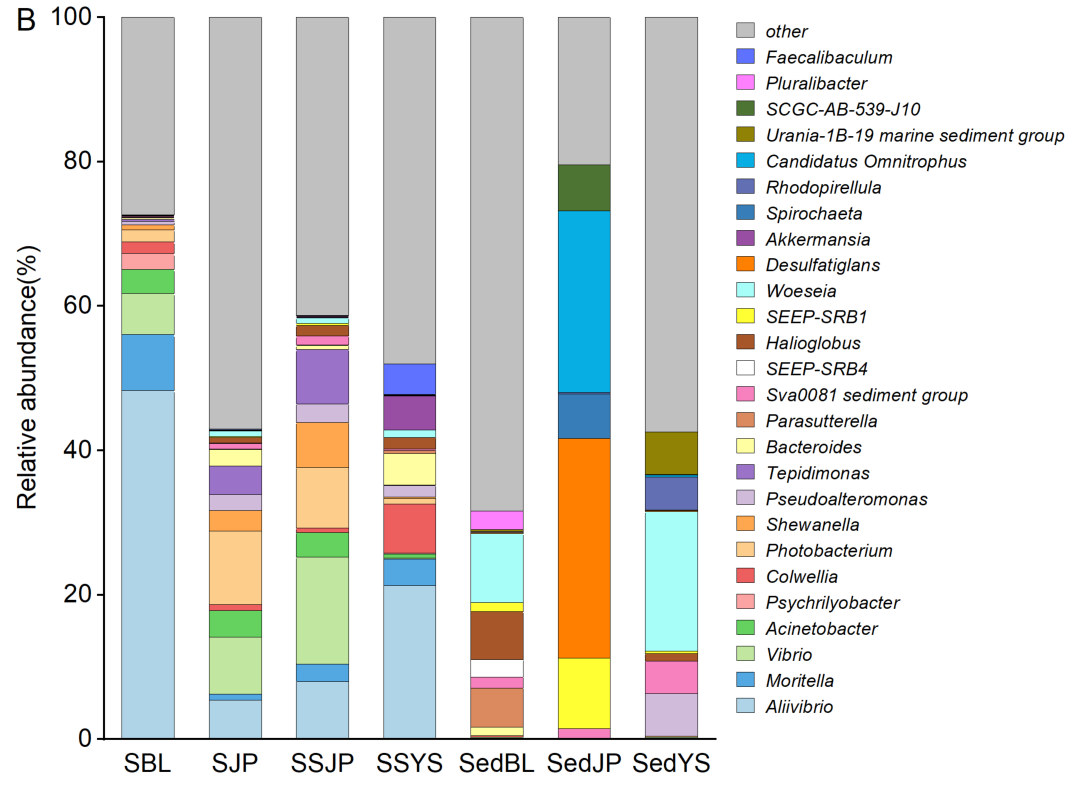


Figure S2 The significant expression pathways of gut microbial community between the *O. sarsii vadicola* from Funka Bay (SSJP) and Yellow Sea (SSYS) on second classification by PICRUSt2 (A) and Tax4Fun2 (B) analysis


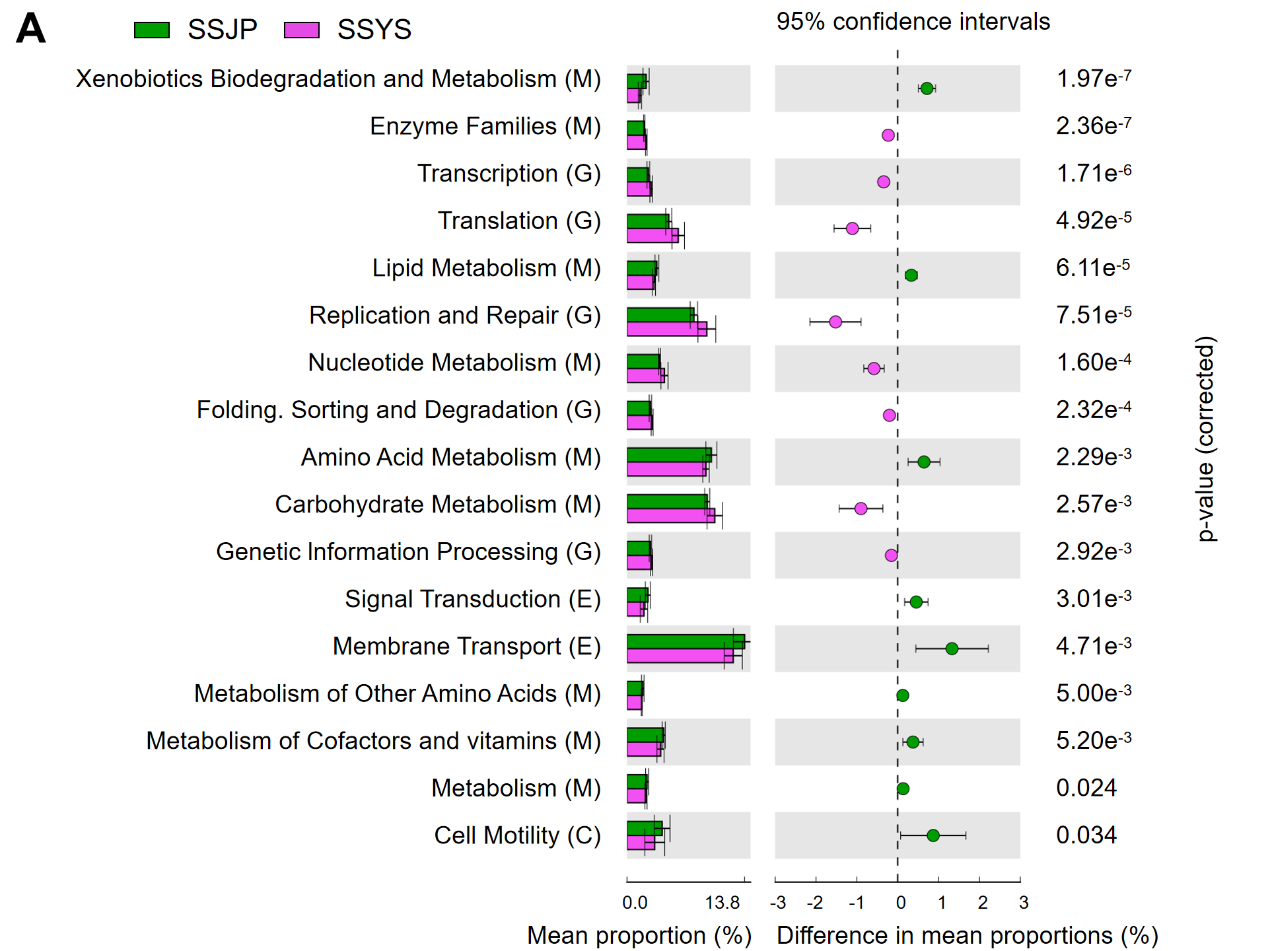

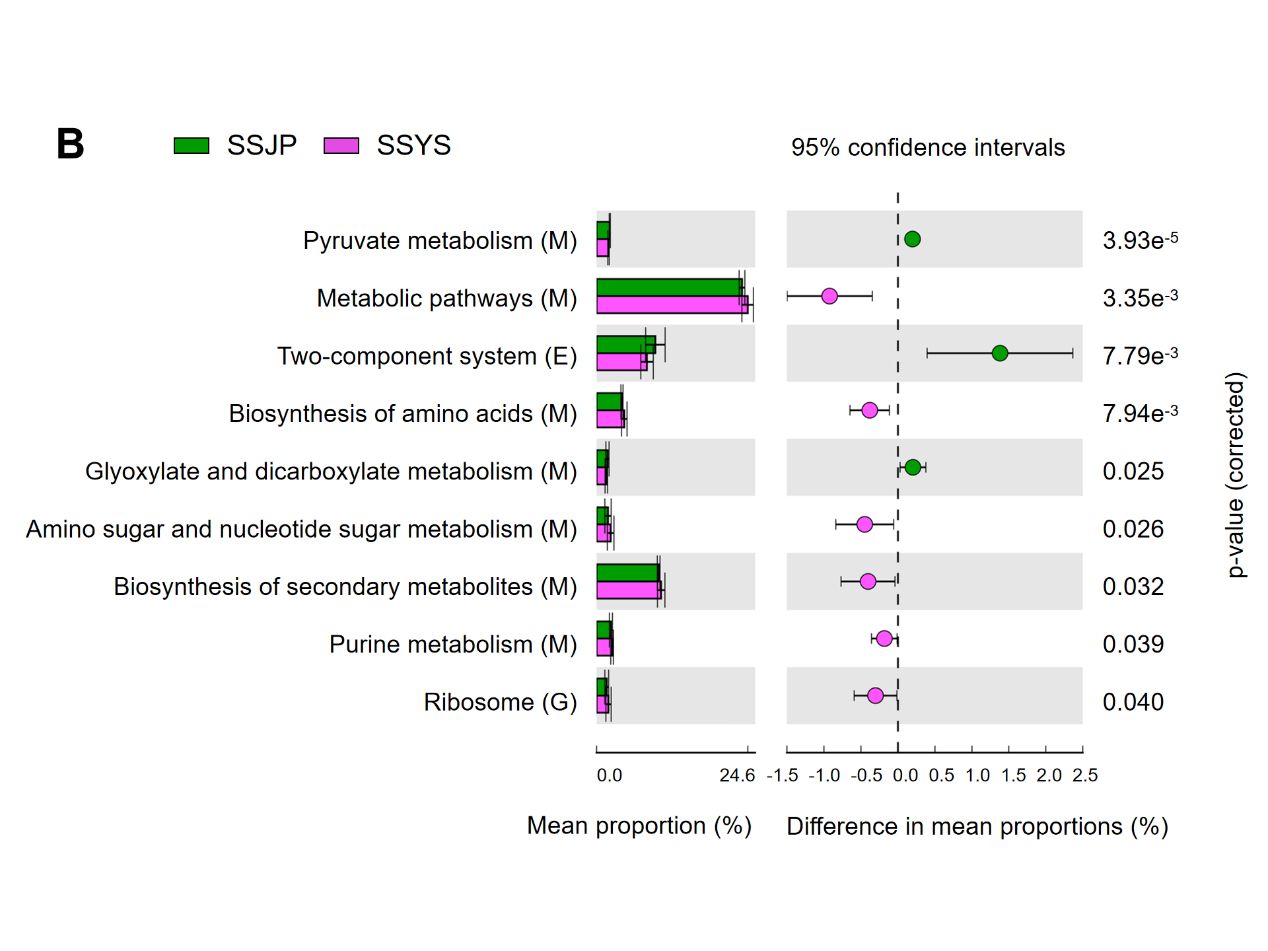


Figure S3 The significant expression pathways of gut microbial community between the *O. sarsii* and *O. sarsii vadicola* from Funka Bay (SJP and SSJP) on second classification by PICRUSt2 analysis


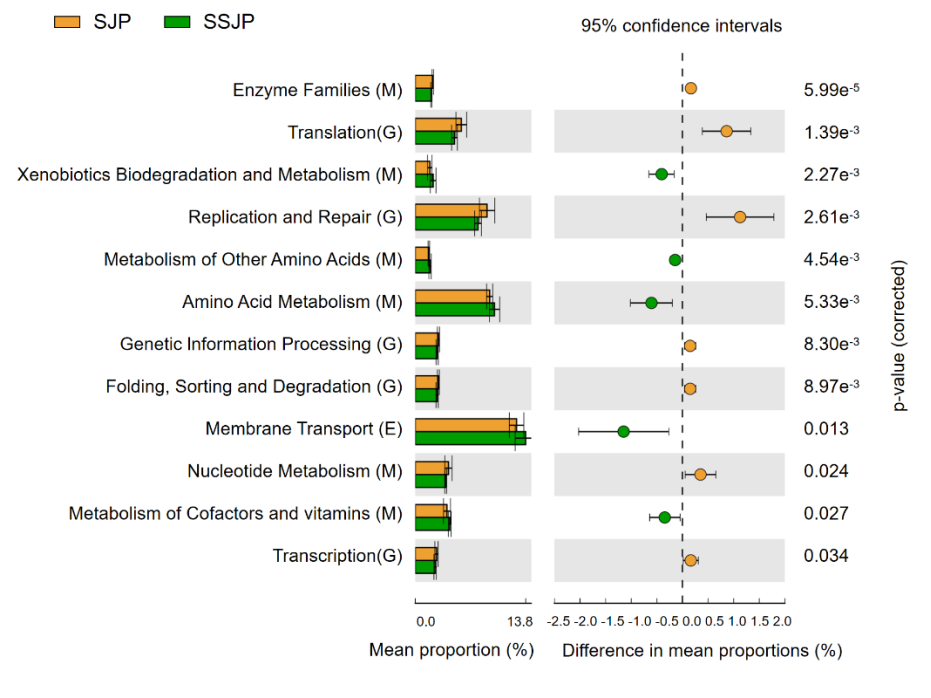


Figure S4 The significant expression pathways of gut microbial community between the *O. sarsii* in Funka Bay (SJP) and *O. sarsii vadicola* in Yellow Sea (SSYS) on second classification by PICRUSt2 (A) and Tax4Fun2 (B) analysis


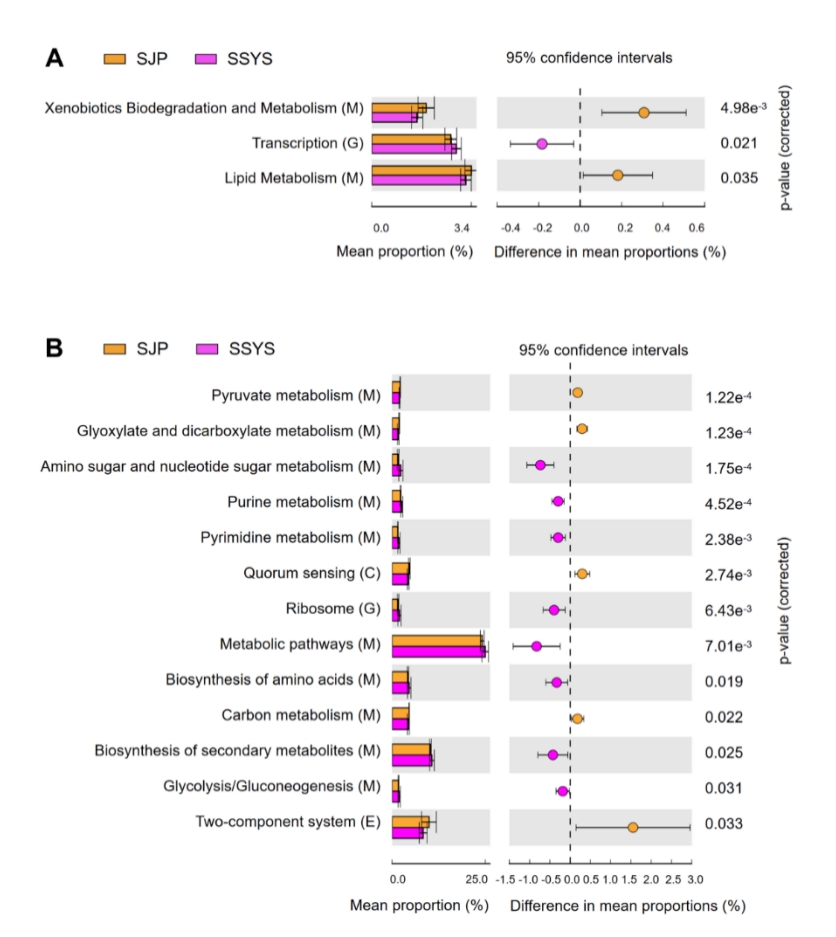


Table S3 The number of genera in different relative abundance range among two group of ophiuroids from Yellow Sea, SSYS was in this study, South YS was in previous study from Dong et al. (2021)

| The range of Relative abundance | SSYS | South YS |
| --- | --- | --- |
| > 10% | 2 | 2 |
| 5 - 10% | 0 | 5 |
| 1 - 5 | 7 | 12 |
| < 1% | 551 | 106 |
| Total | 560 | 126 |
